# A review on anti-nutritional factors: unraveling the natural gateways to human health

**DOI:** 10.3389/fnut.2023.1215873

**Published:** 2023-08-31

**Authors:** Rehana Salim, Iqra Bashir Nehvi, Rakeeb Ahmad Mir, Anshika Tyagi, Sajad Ali, Owais M. Bhat

**Affiliations:** ^1^Division of Food Science and Technology, SKUAST, Shalimar, India; ^2^Sheri Kashmir Institute of Medical Sciences, Srinagar, India; ^3^Department of Biotechnology, School of Life Sciences, Central University of Kashmir, Ganderbal, India; ^4^Department of Biotechnology, Yeungnam University, Gyeongsan, Republic of Korea

**Keywords:** anti-nutritional factors (ANF), nutritional deficiency, plant-based foods, secondary metabolites, human diseases

## Abstract

Humans are constantly facing multiple health challenges from both communicable and non-communicable diseases that significantly affect their health. Additionally, drug resistance or failure has made the situation even worse and poses serious challenges for researchers to develop new drugs. Hence, to address these problems, there is an urgent need to discover and develop timely and long-term-based therapeutic treatments from different sources. One such approach is harnessing the potential of plant secondary metabolites. Plants have been utilized for therapeutic purposes in addition to being used for nutritional benefits. In the last two decades, plant-based drug developments have been one of the effective means of treating human diseases owing to their multiple functions. More recently, anti-nutritional factors (ANFs) have emerged as one of the important targets for novel plant-based drug development due to their multifaceted and potential pharmacological properties. However, their anti-nutritional properties have been the major setback for their limited success in the pharmacological sector. In this review, we provide an overview of ANFs and their beneficial roles in preventing human diseases with multiple case studies. We also highlight the recent developments and applications of ANFs in the food industry, agriculture, and pharmaceutics with future perspectives. Furthermore, we evaluate meta-analyses on ANFs from the last 30 years in relation to their function in human health benefits. This review is an endeavor to reevaluate the merit of these natural compounds and explore their potential for both human and animal health.

## 1. Introduction

Plants and their products have been major sources of nutrients in both human and animal diets. They have also been utilized for therapeutic purposes in addition to being used for nutritional benefits. Biologically active constituents are distributed widely in the plant kingdom, particularly in plants consumed by humans and animals (1). These compounds have both positive and negative effects, depending primarily on their concentration. Among them are anti-nutritional factors (ANFs) or non-beneficial compounds that can affect human and animal growth as well as reduce their nutrient intake, absorption, and utilization. These include phytic acid, saponins, alkaloids, certain oligosaccharides, protease inhibitors, glucosinolates, tannins, and cyanogenic glycosides (2, 3). ANfs are known to alter the absorption of nutrients such as vitamins, minerals, and proteins in addition to inhibiting enzyme activities. There are a plethora of studies that have proven the negative impact of ANFs on nutrient bioavailability in different living organisms (2, 3). However, the deleterious effects of ANFs on nutrient metabolism vary according to age, species, concentration of ANFs, processing, and interactions with other nutrients. Recently, ANFs have attracted considerable interest among researchers owing to their incredible and broad spectrum of biological activities that may be useful to humans (4). For instance, ANFs like saponins are known to possess an array of beneficial effects such as lowering plasma cholesterol levels in humans and possessing anticancer properties, as well as being crucial in reducing the risk of various chronic diseases (5). Similarly, phytic acid has also been shown to be effective in the prevention and treatment of a variety of pathological diseases and cancer through *in vitro* and *in vivo* assays (6). Tannins are polyphenols that, in addition to being antinutrients, have positive effects on humans. Glucosinolates and their companions, such as isothiocyanates (ITCs), on the other hand, have been shown to reduce the risk of cardiovascular and neurological diseases, as well as being anticancer and anti-inflammatory (7). However, there is a glaring information vacuum about how ANFs simultaneously alter food absorption and treat human disorders because earlier studies have mostly concentrated on their detrimental effects on human nutrition and other related features. Indeed, ANFs are potential candidates for future plant-based drug development in humans; however, how they elicit beneficial or detrimental effects on them remains enigmatic at the molecular level. Therefore, more comprehensive biochemical, molecular, and physiological studies are required in different model systems to understand their negative or beneficial roles which will provide novel insights into their implications in human disease therapy. Also, understanding how ANFs contribute to preventing or lowering human disease and the identification of cellular targets or receptors are crucial for the development of ANF-based remedial toolkits for treating human diseases. In this context, integration of multiomics with additional chemical, cellular, drug-designing, and *in silico* methods is necessary to decipher the molecular mechanism and identify the various differentially expressed genes, proteins, metabolites, and ionomes regulated by ANFs in different model systems.

**Figure 1:**
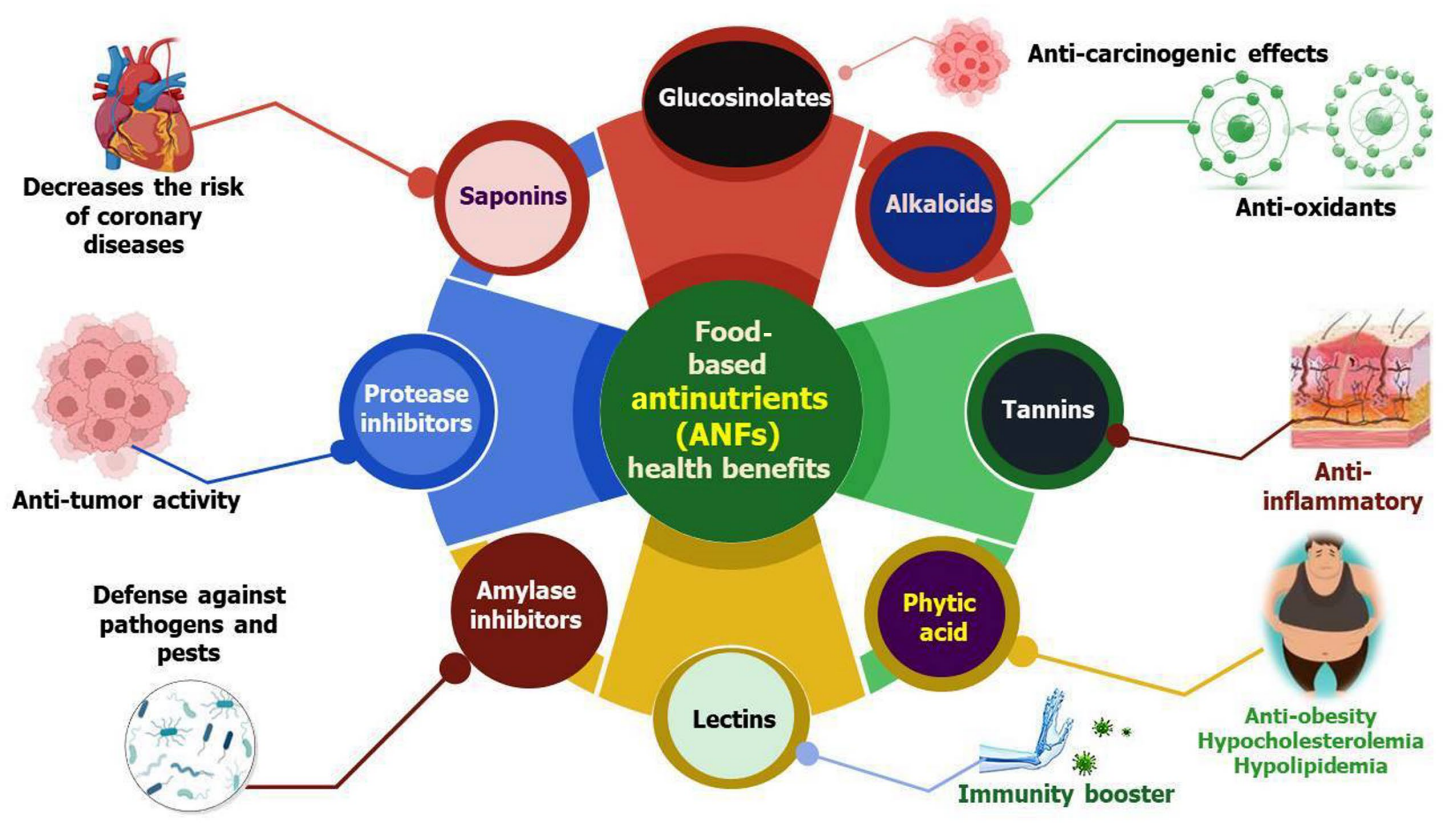
Schematic representation of critical health benefits possessed by ANFs. The figure displays the role of ANFs in the prevention of serious life-threatening human diseases that negatively affect the quality of life. These diseases include cancers, diabetes, bacterial and fungal infections, certain metabolic diseases, hypertension, and cardiovascular ailments.

**Figure 2:**
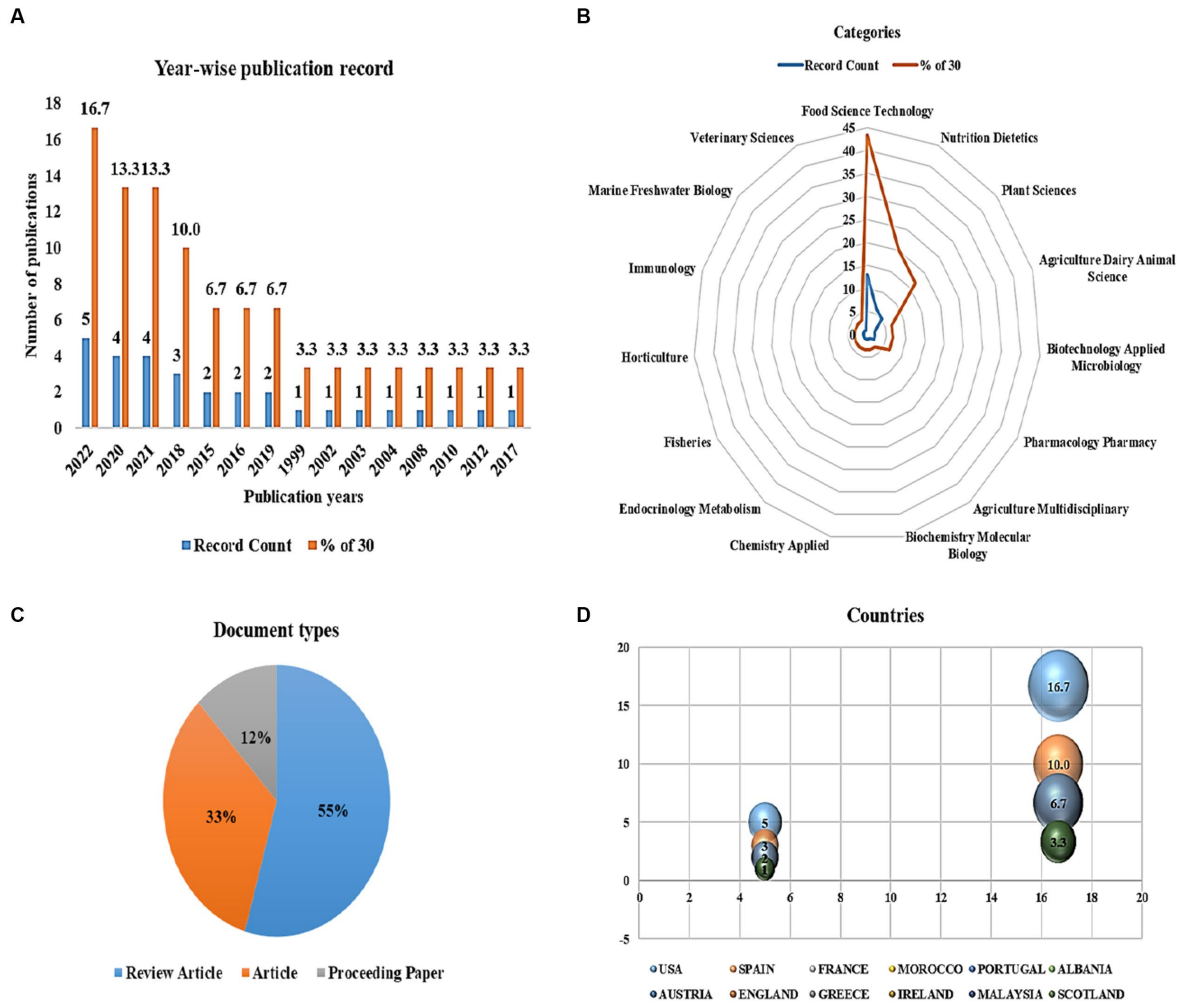
Meta-analyses on the role of anti-nutritional factors and their human health benefits in medical science. **(A)** Year-wise publication records, **(B)** web of science categories, **(C)** document types, and **(D)** countries with the most journal articles published from the past two decades.

ANFs are classified in two ways: as elements that lower nutrient intake in both humans and animals and as compounds that can be found in human or animal diets that affect immunological function and reproductive function (8). They are produced by different metabolic pathways that alter the overall normal nutrition metabolism. The highest levels of antinutrients can be found in legumes, grains, and nuts, but they can also be found in leaves, roots, and fruits of some plant species. ANFs are crucial for protecting plants from herbivores, insects, and diseases, as well as unfavorable growing circumstances (9). Similarly, they can also serve as useful tools to manage various diseases (Figure 1). Previous studies showed that, if consumed in adequate amounts, anti-nutrients can influence nutritional physiology and even act as a natural cure to improve human health (9). A study based on controlled-case trials and epidemiological research reported that many ANFs available in lower concentrations have favorable effects for averting coronary diseases and various cancers [Figure 2; (10)]. For this reason, ANFs are often referred to as plant-bioactive or non-nutritive compounds (2, 11). Many factors influence their activity, for instance, their chemical nature, amount present, and interrelationship with other dietary components. In recent years, a lot of literature has been published on the ANFs of food products which mainly highlighted their negative role in animals or humans. However, the aim of this review is to assess diversified scientific information on the potential health benefits and inimical effects of major ANFs in plant foods. In this review, we provided recent updates on ANFs and their beneficial role in combating human diseases. We also provided an overview of different ANFs found in plants. We also focused on the applications of ANFs in the food industry, agriculture, and pharmaceutics with future perspectives. Further, we examined the meta-analyses on the role of ANFs and their human health benefits in medical science in Web of Science from the past two decades (1999 to 2022) from top-publishing countries for a total number of publication categories and countries in core clinical journals and specific publication types (review articles, articles, and proceeding papers) (Figure 2).

### 1.1. Diversity of anti-nutritional factors and their functions

In plants, different types of ANFs have been found with diverse activities. In this review, we discussed some of the important ANFs and their medical importance. There are several reports on ANFs; however, the majority of them focus on their anti-nutritional characteristics, leaving wide knowledge gaps regarding their beneficial functions. Here, we systematically discuss the beneficial role of ANFs in treating human disorders and also highlight key points for their future research.

### 1.2. Protease inhibitors

Protease inhibitors are ubiquitous in the kingdom Plantae, including in the seeds of legumes and cereals. Protease inhibitors are known to subdue the function of all four classes of proteolytic enzymes, namely, serine, cysteine, aspartyl, and metalloproteinases in the gastrointestinal tract of animals (12–15). Their antinutrient activity is linked to growth inhibition and hypertrophy of the pancreas. In animal studies, it was found that protease inhibitors are associated with pancreatic cancer and, hence, possess anti-carcinogenic effects and a wide range of therapeutic applications [Table 1; (29)]. Protease inhibitors act like competitive inhibitors, i.e., they bind to the active site of the enzyme and form a complex with a very low dissociation constant (10^7^–10^14^ M at neutral pH). The inhibitor imitates the substrate and therefore creates an inhibitor–enzyme complex which cannot be detached through the usual mechanism. This inhibits the active site of the enzyme and, in turn, the protease activity of the enzyme is silenced effectively (30). Inhibitors of serine proteases are the largest group of protease inhibitors. Two large families of protease inhibitors have been identified in legumes: the Bowman-Birk type (BBI) and the Kunitz-type inhibitors. BBI is the most widely categorized family of inhibitors in legume seeds and is composed of small peptide molecules and contains 71 amino acids. These inhibitors contain high levels of cysteine and seven disulfide bridges. The BBI peptide molecules are typically double-headed and so block two protease molecules simultaneously, which may be the same (such as trypsin) or different (such as one chymotrypsin and one elastase or trypsin). The BBI may be resistant to breakdown by heat (31). The Kunitz inhibitors are another class of inhibitors having a molecular weight of approximately 20 kDa. They are peptides of 181 amino acids, commonly found in soya beans and winged beans containing two disulfide bridges. These molecules are single-headed that inhibit the active site of one enzyme molecule (usually trypsin or chymotrypsin) simultaneously (32, 33). According to a recent study, protease inhibitors (PIs) operate as anticarcinogenic agents under *in vivo and in vitro* studies; however, the precise mechanism underlying PIs’ anticarcinogenic effect is not yet fully understood (34). It was found that PIs can affect both the early and later stages of carcinogenesis, but they have no impact on cells that have already undergone transformation (34). PIs have the ability to reverse the initial events, most likely by inhibiting a cellular activity that was initiated by carcinogen exposure. The ability of a PI to affect the expression of specific oncogenes and the amounts of specific types of proteolytic activity is central to its role in the repression of carcinogenesis (34). PIs with antineoplastic action have no discernible effect on normal cells, but they are capable of negating carcinogen-induced cellular changes in different studies (35, 36). The inhibitory profiles of PIs that affect transformation have been examined since not all PIs have the ability to prevent the transformation *in vivo* (37, 38). For instance, chymostatin - a very common and efficient chymotrypsin inhibitor is one of the most effective PI that suppresses the transformation. It has the ability to eliminate radiation–induced transformation *in vitro* with only picomolar concentrations in the medium (38). BBI family inhibitors are effective in suppressing transformation with nanomolar concentrations (39). It has been confirmed that BBIs from soybeans have the ability to inhibit prostate cancer cells’ formation of oxygen radicals and trigger DNA repair through a p53-dependent mechanism (40, 41). Moreover, experiments have shown that BBIs have impeded prostate tumor development by promoting the production of connexin 43 expressions in transgenic rats via its antiproliferative activity (42, 43).

**Table 1:** ANF classes of protease inhibitors, sources, and therapeutic applications.

Name of ANF	Classes/types	Major food sources where ANF is derived	Therapeutic applications	References
Amylase inhibitors	Curcumin 18α-glycyrrhetinic acid Rosmarinic acid Quercetin Phenylthio-ethyl benzoate derivatives Oleanolic Ursolic Betulinic acids	Avocado fruits Pomegranate Noni fruit Olive leaves Cumin	Anti-oxidant Anti-diabetic Anti-obesity Anti-inflammatory Anti-hyperglycemic Anti-hyperlipidemic anti-hepatoprotective	(16) (17) (18) (19) (6)
α-glucosidase inhibitors	Cuminaldehyde Voglibose Flavone-1,2,3-triazole derivatives	Pomegranate Partridge tea Tochucha Welsh onion Cumin	Anti-diabetic Anti-inflammatory Anti-hyperglycemic Anti-hyperlipidemic Anti-oxidant Hepatoprotective Anti-angiogenic effects	(17, 19) (6) (20) (21)
Lectins	Jack bean lectin (Concanavalin A) Soybean lectin Lentil lectins Dioclea lasiocarpa lectins Chickpea lectins Ku Shan and Jack bean lectins *Acacia seyal* Pea recombinant lectins *P. vulgaris* lectin (BTKL) Runner bean lectins	Pseudo-grains: quinoa amaranth, wheat, buckwheat Teff, Nuts: almonds, hazelnut, cashew, pignola, pistachio, brazil nuts, walnuts, macadamia, Chickpea, *P. vulgaris*, Pea	Antiangiogenic Antimetastatic Antiproliferative Antitumoral Antidiabetics Immunomodulatory Antimicrobial	(22–24) (25) (26, 27) (28)

**Figure 3:**
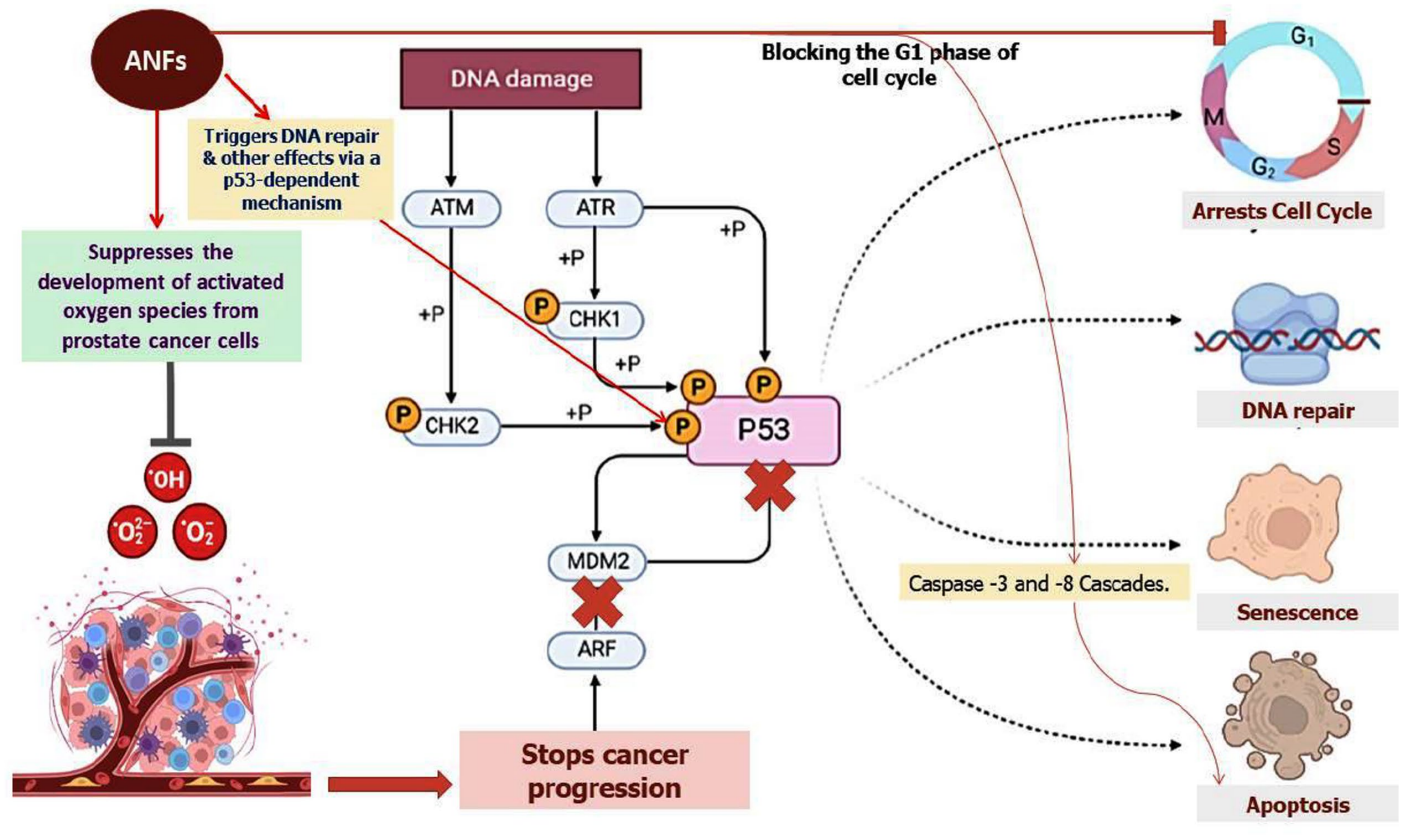
The role of ANFs in altering the cell cycle at the G1 phase. The representation also shows the role of ANFs in repressing the formation of activated oxygen species from prostate cancer cells. ANFs such as TI protein were shown to trigger apoptosis by hindering cell growth and eliciting the courses of caspase-3 and -8 cascades as shown in the figure.

PIs commonly found in raw legume seeds are trypsin and chymotrypsin inhibitors (44). Trypsin inhibitors (TIs) have been connected to increased pancreatic secretory activity, increased pancreatic hypertrophy, and decreased protein digestibility. Additionally, feeding studies including the administration of raw soybeans supplemented with partially purified trypsin inhibitors to rats and chickens resulted in considerable pancreatic hypertrophy and plethoric enzyme secretion (45, 46). Interestingly, a 22 kDa trypsin TI protein from the storage roots of sweet potato (*Ipomoea batatas* L.) has been shown in prior research to have an anti-proliferative activity and works by inhibiting the growth of NB4 promyelocytic leukemia cells (47). According to this study, TI causes NB4 cells to undergo apoptosis by impairing the cell cycle at the G1 phase and activating the caspase-3 and -8 cascades (Figure 3). Trypsin, chymotrypsin, and elastase were all inhibited by protease inhibitors (LC-pi I, II, III, and IV) that were isolated from the seeds of *Lavatera cashmeriana* and were recognized as Kunitz-type inhibitors based on their molecular size (48, 49). Moreover, all four classes of inhibitors exhibited anticarcinogenic activity under *in vitro* conditions. Among all, LC-pi I and II were treated as potential anticancer agents (50). Moreover, under *in vitro* conditions, a strong inhibitory effect of LC-pi I was observed at the initiation stage of cancer in the prostate (PC-3) and breast (MCF-7) cancer cell lines because of the presence of the protease inhibitor activity of trypsin, chymotrypsin, and elastase (49).

## 2. Amylase and α-glucosidase inhibitors: a natural gateway to cure hyperglycemic patients

**Figure 4:**
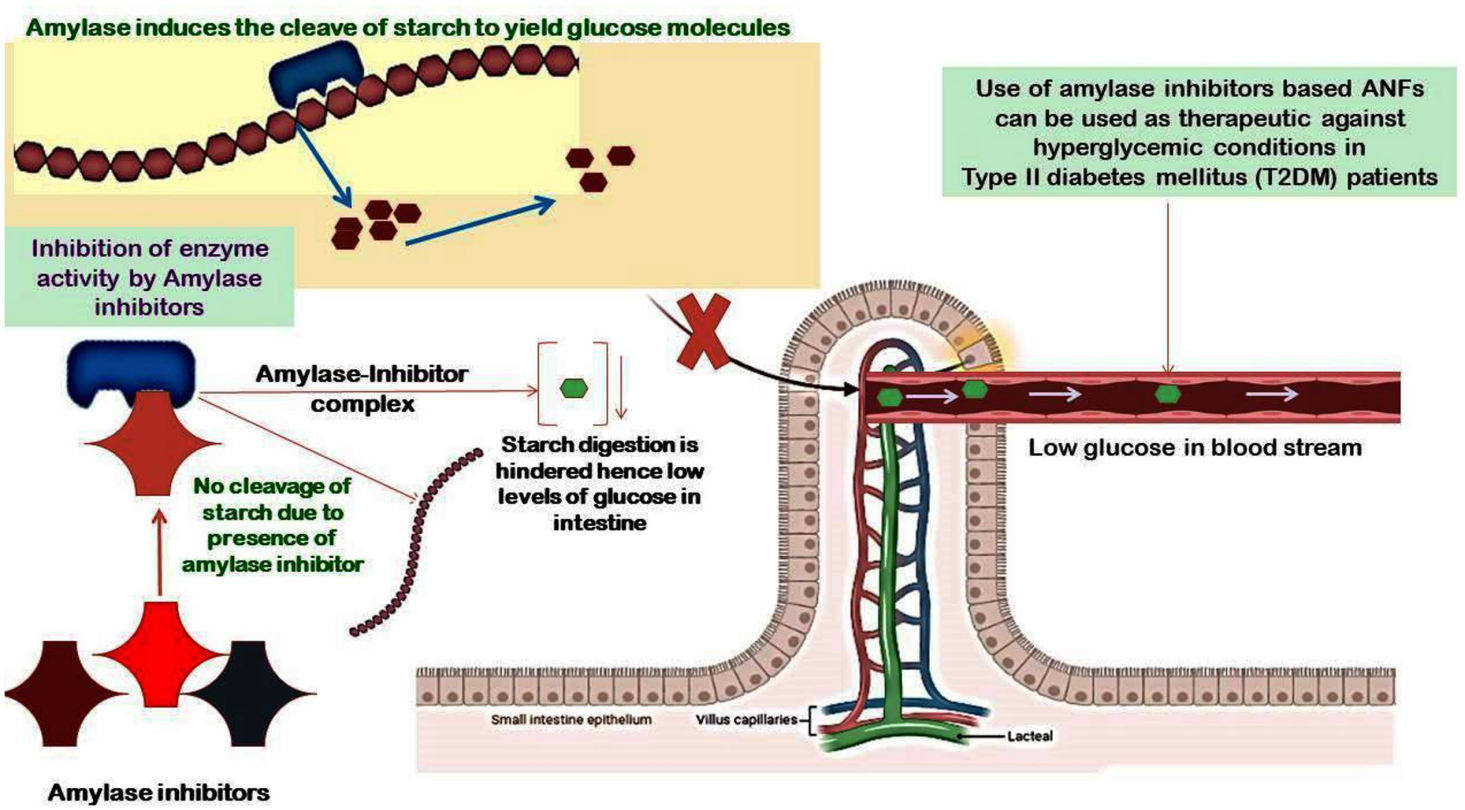
Potential targets of food-based amylase class of ANFs on the prevention of hyperglycemic conditions in diabetic patients. The α-amylase inhibitors in whole food grains are usually released during the process of digestion in the GI tract, and these inhibitors form a complex with the α-amylase, hence blocking its activity to limit the hydrolysis of starch and lowering the production of glucose molecules. This mechanism paves the way for amylase inhibitors-based therapeutics in individuals suffering from diabetes to maintain a low glycemic index.

Many plants have amylase enzyme inhibitors, which are important for controlling endogenous-amylase activity as well as defense against pathogens and pests (51). They are essential for humans because they help convert dietary carbs into glucose molecules, which prevent the body from absorbing dietary starches. They are commonly found in pigeon peas and effectively function within a pH range of 4.5–9.5 (52). Amylase inhibitors are known to be heat-labile and decrease the action of bovine pancreatic amylase, but they have no effect on the activity of bacterial or fungal amylase. The use of natural enzyme inhibitors is of dire requirement due to the large side effects imparted by synthetic enzymes. Inhibitors such as derivatives from phlorotannin including fucodiphloroethol G, dieckol, 6,6′-bieckol, 2-phloroeckol, 7-phloroeckol, phlorofucofuroeckol A, 2,7′-phloroglucinol-6,6′-bieckol, 2-O-(2,4,6-trihydroxyphenyl)-6,6′-bieckol, 8,8′-bieckol, 6,8′-bieckol, and eckol possess strong inhibitory activity against α-amylase and α-glucosidase (53–55). All these inhibitors have been reported to be potential therapeutic agents against diabetes, and they have been recorded as possessing hypoglycemic effects. However, their unsteadiness in the gastrointestinal pathway led to the failure in reduction of insulin response and enhanced the caloric output of victuals by adopting them as starch-blocker pills. The α-amylase inhibitor from white beans (*Phaseolus vulgaris*) has been reported to potentially induce weight loss and lower blood sugar levels brought on by a diet high in carbohydrates (56, 57). Furthermore, renin and angiotensin-I-converting enzyme activity is inhibited by the penta- and hexapeptides produced from pigeon peas, which also have strong antioxidant characteristics (58). Amylase inhibitors are attributed to possessing two functions: protecting the seeds from microbial infections and pests and also inhibiting endogenous amylase (59). The inhibitors of α-amylase play a critical role in treating type-2 *Diabetes mellitus* by slowing down the absorption of glucose molecules in the body (60, 61). Moreover, fucoidan derived from several species of the genus *Sargassum* is shown to possess anti-diabetic activity by inhibiting the enzymes α-amylase and α-glucosidase (62–69). Moreover, several inhibitors such as octaphlorethol A (OPA), diphlorethohydroxycarmalol (DPHC), and ishophloroglucin A (IPA) derived from *Ishige okamurae* and *Ishige foliacea* possess strong inhibitory activity against α-amylase (70, 71). The latter may serve as possible medications to treat non-insulin-dependent *D. mellitus*. Plant-based phytochemicals comprising of phenol are artless inhibitors of α-amylase and α-glucosidase with a much stronger effect on α-glucosidase and moderate repressive impact on α-amylase. Thus, with little side effects, these phenolic phytochemicals can be employed as an effective strategy for preventing postprandial hyperglycemia (72). Equally, consuming a diet high in mixed carbohydrates and inhibiting α-amylase and α-glucosidase through the action of phenolic antioxidants will minimize postprandial hyperglycemia and may be a successful method of managing type-2 diabetes. Flavan-3-ols are believed to intervene in the incipience of cardiovascular disease through different mechanisms such as antioxidative, anti-thrombogenic, and anti-inflammatory processes. Proanthocyanidins (PAs) and flavan-3-ol monomers, specifically, contribute to reducing the cholesterol levels in blood plasma, prevent LDL oxidation, and activate endothelial nitric oxide synthase to avoid platelet adhesion and aggregation that can lead to blood clot formation (73, 74). All these reports clearly indicate that amylase inhibitors have great potential in treating diseases, especially type I diabetes, and the findings will pave the way in the design of dietary guidelines that can aid in maintaining good human health. Further, we have shown the potential targets of food-based amylase class of ANFs on the prevention of hyperglycemic conditions in diabetic patients (Figure 4).

## 3. Lectins

Proteins that can conglomerate RBCs with specified sugar selectivity are referred to as “lectins” (75). They are also called hemagglutinins when the sugar specificity is not known. Lectins are glycated proteins extensively present in legumes and certain oil seeds such as soya beans (76). They possess at least one non-catalytic domain, which is reversibly bound to monosaccharides or oligosaccharides. They can agglutinate the erythrocytes by binding to the carbohydrate moieties present on the surface of erythrocytes without modifying the carbohydrate properties (77). Lectins can damage the intestinal cells by binding to intestinal epithelial cells and impair nutrient absorption leading to infiltration of bacteria into the blood stream (78). Lectins can bind to RBCs, causing hemagglutination and anemia. Lectins are the hot spot for biologists, especially for agricultural and medical research applications (77). Studies have revealed new evidence of potential light in lectins, particularly cereal lectins in the etiology of human disorders like rheumatoid arthritis and cardio vascular diseases (CVDs) (79). Plant Lectins administered orally may have an extensive impact on varied types of tumors. For instance, a study showed that galactose-specific peanut agglutinin, PNA, activates cell proliferation in colonic explants *in vitro*, and eating peanuts enhanced rectal proliferation in persons with a mucosal expression of the peanut lectin receptor (80). It has been reported that the lectin from kidney beans prevented the development of tumors in NMR and BaLB/c mice (81). Many lectins are employed as therapeutic agents because they preferentially bind to cancer cell membranes or their receptors, producing cytotoxicity, apoptosis, and prevention of tumor growth. These lectins have been proven to have anticancer characteristics *in vitro, in vivo*, and in human case studies (80, 81). Lectins have the potential to enter cells and cause cancer cell agglutination or aggregation. The beneficial impact of lectin is complex due to a number of factors including dispossession of nutrients of the growing tumor by the altitudinous nutrient and polyamine *sine qua non* for lectin-induced compulsory gut growth, suppression of angiogenesis by kidney bean lectin in the growing tumor, and stimulation of the immune system to counter the tumor growth. Consequently, lectins have great potential to act as therapeutic molecules within limited dose and route.

## 4. Naturally derived ANF compounds

Several other plant-derived ANFs have been reported to possess both beneficial and toxic effects on human health. For instance, certain compounds interfere with the solubility or absorption of minerals. These compounds include glucosinolates, phytic acid, oxalic acid (thioglucosides), and gossypol. In general, plant-derived compounds have been attributed to several therapeutic applications (refer to Table 2). These compounds include tannins, catechins, phytic acid, glucosinolates, saponins, and alkaloids. In this context, we have detailed the therapeutic applications of these ANFs in the preceding sections of this review.

**Table 2:** ANF classes of natural chemical compounds, sources, and therapeutic applications.

Name of ANF	Classes/types	Major food source from where ANF is derived	Therapeutic Applications	References
Tannins	Phlorotannins- Eckol Dieckol 6,60-bieckol Phloroglucinol Phlorofucofuroeckol A Triphlorethol-A Eckstolonol, Fucosterol Octaphlorethol A	Chickpea, walnuts, grapes, plums, cocoa, coffee, raspberries, lentils, sea weeds, Pseudo-grains: quinoa, amaranth, wheat, buckwheat, Teff, Nighshades: potato, tomato, eggplant, Pepper	Anti-mutagenic Chemopreventive Antimicrobial Cardioprotective Antioxidant Anti-cancer Anti-bacterial Anti-allergic Anti-diabetic Anti-inflammatory Anti-hypertensive Anti-obesity	(82) (83–85) (86) (87–89) (87)
Catechins	Epigallocatechin-3-gallate β-sitosterol Lignin glycosides Epigallocatechin gallate	Green Tea Ashoka tree Kodo Millet Little Millet	Anti-microbial Anti-cancer Antioxidant Anti-inflammatory Anti-Melanogenesis Anti-Adipogenesis Prevention of skeletal muscle atrophy	(90) (3) (91) (92) (93) (94)
Phytic acid		Grains- wheat, barley, rye, oat, millet, corn, spelt, kamut, sorghum, Seeds: sesame, flaxseed, poppy seed, sunflower, pumpkin, Nuts: almonds, hazelnut, cashew, pignola, pistachio, brazil nuts, walnuts, macadamia, Nighshades: potato, tomato, eggplant, Pepper	Antioxidant Humans Anticholesterolemic Antidiabetics Neuroprotective Chemopreventive Antiosteoporotic	(95) (96) (97) (98) (99–101) (102)
Glucosinolates	Sinigrin Gluconapin Glucobrassicanapin Progoitrin Glucoibervirin Allyl isothiocyanates (AITCs) Goitrogens Thioglucosides	Cabbage, mustard, rapeseed green, turnips, rutabaga,	Antiproliferative Chemopreventive Anticholesterolemic/ Antiinflammatory Antiasthmatic Neuroprotective	(103) (104) (105) (106) (107)
Saponins	Diosgenin, protodioscin, β-diglucorhamnoside, saikosaponins,	Legumes- soya, lentils, chickpeas, peanuts, beans, Nighshades: potato, tomato, eggplant, Pepper	Anticancer bioactivity Anti-inflammatory cardiovascular diseases Anti-bacterial Anti-oxidative	(108) (109) (110) (111) (112)
Alkaloids	Phyto-Carbazole Rutaecarpine Dihydroberberine 3-methylcarbazole		Protective Agents against Neurodegenerative Diseases Antioxidant Anti-cancer Cell cycle arrest Anti-Inflammatory Anti-viral activity Treatment of ulcerative colitis Anti-aging Anti-diabetic	(113) (114) (110) (115) (116) (117) (118)

### 4.1. Tannins: a functionally multifaceted ANF for enhancing human health

Tannins are polyphenolic substances with molecular weights ranging from 500 to 3,000 daltons. Chemically, they are classified as condensed tannins/pro-anthocyanidins and hydrolyzable tannins (119). Hydrolyzable tannins (HT) are compounds with a central polyol (D-glucose) core. The hydroxyl groups in these carbohydrate molecules are in part or completely esterified with phenol moiety-containing compounds, such as gallic acid (gallotannin) or ellagic acid (ellagitannin). HTs frequently occur in less quantity within plants and are easily hydrolyzed by mild acids and bases into carbohydrate and phenolic acids. Condensed tannins are naturally present in polyphenolic bioflavonoids, typically in the conformation of oligomers or polymers of polyhydroxy flavan-3-ol units, like (+)-catechin, (−)-epicatechin, flavan-3,4-diols, leucoanthocyanidins, or a combination of the two (120). Condensed tannins are commonly present in tree barks as barriers against microbes and are also found in other tissues of plants including buds, leaves, roots, seeds, and stems. The anti-nutritional effects of tannins are determined by their chemical conformation, dosage, and tolerable daily intake (29). Tannins have been documented to affect the digestibility of proteins and influence the bioavailability of non-heme iron, leading to less absorption of iron and calcium. They have an effect on carbohydrate digestibility, contributing to the reduced energy value of a diet (121).

Tannins are known to bind proteins through hydrogen bonds and hydrophobic interactions. They are known to interact with digestive enzymes, which exhibit anti-trypsin and anti-amylase activities, thus rendering them unavailable for digestion. It has been confirmed that condensed tannins triggered the testa-bound trypsin inhibitor activity (45). Tannins can also form a complex with vitamin B. They can also contribute to decreased palatability and lower growth rate (76). The implication of food tannins on human health is a common worry although they do have some beneficial effects as well. Some tannins possess antioxidant activity and are considered to be cardio-protective, anti-inflammatory, anti-carcinogenic, and anti-mutagenic (122, 123). Tannins are reported to inhibit lipid peroxidation and lipoxygenases under *in vitro* conditions. Recent studies indicate they have the ability to scavenge radicals, such as hydroxyl, superoxide, and peroxyl, which are considered to be important cellular prooxidants (124). According to an *in vitro* study using the human colon cancer cell line HT29, proanthocyanidin-rich apple polyphenol extract prevented the phosphorylation of epidermal growth factor (EGF), which in turn suppressed the development of these cells (125). Additionally, it has been shown that these substances inhibit the growth of cancer cells under *in vivo* conditions (126). Moreover, it is reported that tannins have the potential to enhance glucose absorption and prevent adipogenesis (127, 128). A class of tannins known as phlorotannins has been found to provide significant health benefits, including anti-oxidant, anti-inflammatory, anti-cancer, anti-proliferative, anti-bacterial, anti-mutagenic, anti-allergic, anti-diabetic, anti-obesity, and anti-hypertensive properties (83, 86–89). For instance, phlorotannins such as pyrogallol-phloroglucinol-6,6-bieckol, dieckol, 2,7-phloroglucinol-6,6-bieckol, and phlorofucofuroeckol A profoundly inhibit the dysfunction of suppressing monocyte migration, death of monocyte-associated endothelial cell, and inhibition of inflammation by monocyte-associated vesicles (129). All these studies strongly suggest that tannins are potential therapeutic agents against a wide range of diseases. Henceforth, it is critical to investigate the proper protocol for standardizing the dosage and troubleshooting its therapeutic effects through *in vitro* and *in vivo* studies.

### 4.2. Catechins

Catechins (flavan-3-ol) are a group of secondary metabolites of flavonoids that have two benzene rings and a dihydropyran heterocycle. They are known to protect both involuntary and radiation-induced cancers as well as chemically generated mutations. Catechins promote phase I and II metabolic enzymes that enhance the formation and excretion of detoxified carcinogenic metabolites, inhibit cell division, and, thus, slow the emergence and spread of cancer. Catechins are strong antioxidants that prevent the oxidation of LDL cholesterol, lower cholesterol, and reduce body fat, all of which help to lower the risk of cardiovascular disease (122). Moreover, they help to reduce high blood pressure-induced strokes (122). Catechins and polyphenols present in tea leaves are efficient scavengers of reactive oxygen species (ROS) and act as indirect antioxidants by influencing transcription factors and enzyme activities. In humans, mild improvements in plasma antioxidant ability were observed following the consumption of catechins from green tea. Catechins present in tea and green tea have a very positive effect on the biomarkers of oxidative stress, particularly on the oxidative damage caused to the DNA in animal models, but human data on biomarkers of *in vivo* oxidative stress is limited. Greater human research studies examining the impact of tea and the catechins present in them on the biomarkers of oxidative damage to lipids, proteins, and DNA are required (130). Owing to its multiple therapeutic properties, it is vital to investigate the role of catechins in driving complex networks and unravel the underlying mechanisms to regulate the wide range of disorders.

### 4.3. Phytic acid

Phytic acid is a myoinositol 1,2,3,4,5,6-hexakis dihydrogen phosphate found in plants, animals, and soil in the form of a salt of the mono and divalent cations K^+^, Mg^2+^, and Ca^2+^. It is the major cache of phosphorous encompassing 1–5% by weight in cereals, legumes, oil seeds, and nuts (131). Phytate promptly proliferates in the seeds during their ripening period. They are hoarded within leguminous seeds and oil seeds inside the globoid crystal in the protein bodies. Phytates also serve as reservoirs of cations, which are high-energy phosphoryl groups, and by chelating free iron, act as an efficient natural antioxidant (132). Monogastric animals like poultry and humans cannot break down phytic acid due to the absence of an adequate quantity of phytate-reducing enzymes in their alimentary tract (133–135). Phytates act as potent anions in a broad pH range and thus have an unfavorable impact on the bioavailability of divalent and trivalent mineral ions such as Zn^2+^, Fe^2+/3+^, Ca^2+^, Mg^2+^, Mn^2+^, and Cu^2+^ in diet (29). The impact of an increased intake of a phytate-containing diet on mineral deficiency depends on what else is being consumed. The intake of phytate is a problem in regions of the world where cereal proteins are a significant and preponderant dietic component (25). Phytates substantially reduce the bioavailability of calcium, and the molar ratio of Ca^2+^: phytate has been recommended as a marker of Ca^2+^ bioavailability. The critical molar ratio of Ca^2+^: phytate is specified to be 6:1 (136). It has been reported that the interaction between phytate and carbohydrates (starch) reduces the bioavailability and breakdown of carbohydrates as the formation of the phytate-carbohydrate complex affects the rate of metabolism of starch (137). Nonetheless, recent data reveals positive health effects of phytic acid on hypocholesterolemia and hypolipidemia as an anticancer agent. The prophylaxis and suppression of tumor evolution and progression are primarily associated with the ability of IP6 to regulate the segregation, multiplication, and programmed cell death of tumor-forming cells (138, 139). This IP6-conferred anticarcinogenic protection is associated with its suppression of the generation of oxygen-free radicals by the preclusion of the Fenton reaction in iron chelation (140). Reports suggest that phytate inhibits the growth of human cell lines such as leukemic hematopoietic K-562 cell line (141), colon cancer HT-29 cell line (142), breast cancer cell lines (143), cervical cancer cell lines (144), prostate cancer cell lines, and HepG2 hepatoma cell line (145) in a dose- and time-dependent manner. Nonetheless, the phytate sensitivity of cells from different origins varies, indicating that phytate can affect different cell types by specific mechanisms of action. Phytates’ effectiveness as an anticancer agent was also shown in carcinogen DMBA-induced breast cancer rats. The animals treated with phytate showed a significant decline in proliferation. The study also compared the oncogenic indicators like serum total sialic acid (TSA), and documented grades of tissue nitric oxide to reflect carcinoma activity after administration of DMBA. Phytic acid administration in comparison to the control group considerably decreased the generation of TSA, increased programmed cell death, and suppressed oxidative stress linked with the generation of oxygen-free radicals from the carcinoma, therefore implying a probable medicinal value of phytic acid in breast oncogenesis. Phytic acid reduces the rate of cell multiplication in breast carcinoma, functions as an antioxidant, and also enhances programmed cell death (138). Dietary phytate may also aid diabetic patients as it suppresses the blood glucose levels by reducing the rate of metabolism of starch, hence slowing gastric emptiness. Similarly, phytate has also been proved to modulate the release of insulin (146). Across the western countries, cardiovascular disease is considered a leading cause of death. Elevated plasma cholesterol or elevated LDL-cholesterol concentrations have been shown to be one of the risk factors of heart diseases. Phytate, which is a component of fiber, is believed to affect the etiology of heart diseases. It has been shown that the reduction in serum cholesterol and triglyceride levels arises as a result of dietary phytate supplementation (147, 148). The decrease in serum Zn level and Zn-Cu ratio was correlated with this effect, and an imbalance in Zn-Cu metabolism is related with coronary heart disease. Phytic acid has been accepted as a superior preservative for juices (149) and meat products (150, 151). In addition, apple extract tempered with IP6 during distillation and packaging have exhibited a substantial diminution in the discoloration (brown color formation) due to polyphenol oxidase inhibition by IP6, whereas pigs fed with diet containing IP6 exhibited an enhanced shelf life of meat. The impact of IP6 on unsaturated fatty acids helped in maintaining the quality of meat and also enhanced the shelf life by suppressing lipid peroxidation (150–152). All these studies clearly approve the therapeutic potential of phytic acid and hence demands further investigation to unravel its proper use and applications.

### 4.4. Glucosinolates

Glucosinolates are an important class of phytochemicals present in *Brassica* vegetables within the range of 1.5–2.0 mg/g. They are primarily found in cabbage, broccoli, and Brussels sprouts. (153–155). Most glucosinolates are chemically and thermally stable but their hydrolytic derivatives are physiologically active (154). In raw vegetables, enzymatic hydrolysis occurs when cells are disrupted by masticating or refining the release of β-thioglucosidase (156). Glucosinolates are digested by microflora in the human GI tract and can therefore be utilized biologically in cooked vegetables even though cooking vegetables inactivates thioglucosidase. (153, 157). When plant tissue is damaged, the enzyme thioglucosidase breaks down the thioglucosidic bond, resulting in the creation of glucose and thiohydrosimate-O-sulphonate (an unstable aglycone) (155, 156). Depending on the reaction parameters such as pH and glucosinolate structure, different products can be obtained including isothiocyanates, nitriles, sulfides, thiocyanates, epithiitriles, oxazolidin-2-thiones, and indolyl compounds (154, 156). Hydrolytic decomposition products of glucosinolates, glucoraphanin, gluconasturtiin, and glucobrassicin exhibit antioncogenic properties. Furthermore, indol-3-carbinol, a component of glucobrasicin has the ability to inhibit human breast and ovarian cancers (158–160). Vegetables from the *Brassica* family comprise thioglycosides that are broken down into thiocyanates, which, in turn, impede the transport of iron and integration of iodine in thyroglobulin, ameliorating the augmentation of TSH secretion and thyroid cells. There is little epidemiological proof that the goitrogenic effects of glucosinolate degradation products lead to significant causes of human disease. There is significant doubt about the positive and negative repercussions of *Brassica* vegetables on health since there are major concerns regarding the breakdown products of glucosinolate and their antioncogenic effects. (161). Over 130 different glucosinolates have been identified as having antioxidant, anticancer, fungicide, and bactericidal properties (162, 163). For instance, isothiocyanates decrease redox imbalance levels in the system, alter chemokine function based on the reaction of the immune system, prompt programmed cell death, hinder the advancement of the cell cycle, prevent the formation and differentiation of blood vessels, and also show anti-bacterial, anti-viral, and anti-carcinogenic properties (160, 164–166). There are primarily two routes that have been proposed for isothiocyanates’ anticarcinogenic effects. First, phase II enzymes are activated concurrently with the inactivation of the phase I enzyme cytochrome P450s, which binds to isothiocyanate. Another mechanism involves the start of programmed cell death, which eliminates genetically compromised cells and halts the cell cycle (167–169). It has been proven that sulphoraphane from broccoli inhibits the growth of tumors by acting as the main inducer of cell-defensive phase II enzymes. As broccoli and Brussels sprouts are highly consumed, the extracts obtained from them are regarded as an appropriate tool for supplying sulphoraphane to humans (170). In another study, it was shown that 4-methylthiobutyl isothiocyanate specifically induced cytotoxicity in tumor-initiating cells via the p53-independent pathway; however, no apoptosis or necrosis was detected when used on normal liver cells. This compound was produced by the enzymatic breakdown of glucoerucin isolated from plants of the rocket species or by catabolism of sulphoraphane of isothiocyanate (165). In a clinical trial, the consumption of 250 g/day of broccoli and 250 g/day of Brussels sprouts significantly increased the clearance of the potentially cancer-causing chemical 2-amino-1-methyl-6-phenylimidazo [4,5-b] pyridine (PhIP) found in properly cooked meat. It has been demonstrated that excess intake of vegetables from the *Brassica* family can minimize the risk of colorectal cancer by augmenting the excretion of PhIP and diet-containing heterocyclic amine carcinogens (171).

### 4.5. Saponins

Saponins are non-volatile surface-active secondary metabolites that are typically found in plants. They are made up of a sugar moiety coupled with a steroid (or triterpene) group. These surface-active compounds are present in legumes in addition to some spices and herbs (172, 173). High concentrations of saponins impart astringency and a bitter flavor to food plants. They have historically been considered to be antinutrients due to their negative effects, for instance, growth degradation and their bitter taste, and their throat-irritating nature has led to their reduced consumption and is a key limiting factor in their application (174). Saponins have been shown to decrease the physiological availability of nutrients and enzymes and they also hinder the activity of certain metabolic catalysts such as trypsin and chymotrypsin and thus affect protein digestibility (175). Recent studies demonstrate saponins’ ability to lower cholesterol, boost the immune system, and prevent cancer (176). They also decrease the peril of coronary heart disease in humans. Foods rich in saponin are essential for regulating the level of cholesterol in blood plasma, preventing peptic ulcers and osteoporosis, and decreasing the risk of heart diseases (177). Saponins are used as adjuvants in viral (e.g.*, Quillaja saponaria-*21) and bacterial vaccine (e.g.*, Quillaja* saponins) applications (174). A diet high in saponins can be used to treat acute lead poisoning, prevent dental cavities, inhibit platelet aggregation, and cure hypercalciuria in people (178).

### 4.6. Alkaloids

Alkaloids are secondary metabolites found in a wide range of plant species that possess therapeutic properties. Alkaloids, which primarily represent themselves as specialized molecules participating in metabolism, are nitrogen-containing chemicals having the capacity to react with acids to produce salts (179). Alkaloids appear to be present in distillate from roots, seeds, leaves, or barks of affiliates of at least 40% of plant families. Families including Amaryllidaceae, Compositae, Leguminosae, Liliaceae, Papaveraceae, and Solanaceae are especially rich in alkaloids. Solanine and tomatine are common examples. Solanine is found in little concentrations in potatoes, whereas tomatine is present in tomatoes. Alkaloids are premeditated to be anti-nutrients due to their effect on the nervous system, hindering or erroneously enhancing electrochemical transmission. The glycoalkaloids, solanine, and chaconine found in potato and Solanum spp. (180) are hemolytically active and harmful to fungi and humans. There have been studies on the infertility effects of certain plant alkaloids (181) although less intake of alkaloids arbitrate pivotal therapeutic functions, such as pain reduction, blood pressure control, tumor cell destruction, and stimulation of circulation and respiration (175).

## 5. Applications of ANF in the food industry, agriculture, and pharmaceutics

ANFs are generally considered harmful biologically active compounds due to their negative effect on nutrition availability and absorption. However, recent studies have shown that they have an incredibly beneficial role in preventing human diseases, and hence have become one of the important targets for plant-based drug development. However, plant-based diet also contains an array of ANFs or non-beneficial compounds that can affect human and animal growth as well as reduce their nutrient intake, absorption, and utilization. These include phytic acid, saponins, alkaloids, certain oligosaccharides, protease inhibitors, glucosinolates, tannins, and cyanogenic glycosides. Refined saponins or their concentrates have been used as additives in the production of food and beverages mainly as foaming agents or as emulsion stabilizers. In addition to foods, saponins are also used as anti-oxidants (182). Saponins are utilized in the manufacturing of lyophilized powders with vitamin E for the enhancement of foods, drinks, and animal feeds. The benefit of isoflavones is due to their oestrogenic activity and possible application as enhancers of growth in the fodder industry (183). Flavonoids have been shown to prevent thermal or chemically caused lipid peroxidation as well as chelating metallic and super oxide ions in the food processing industry (184). Flavones and leucoanthocyanidins impart scrumptious zing to the foods after processing (185). Using flavones as additives in many soft drinks and lemon brands imparts a peculiar bitter taste to them. The suppression of the growth of many plant species by saponins from alfalfa and *Vernonia amygdalina* (Compositae) leaves have been reported (186–188). Flavonoids are also known to be used as seed germinators and plant growth regulators. Tannins have also been known to be useful in forages (189). It has also been suggested that animal food with relatively higher amounts of proanthocyanidins (2–4% digestible matter) affects protein metabolism in a positive way, delaying the breakdown of dietary proteins into ammonia by micro-organisms present in the rumen and improving the outflow of protein from the rumen, thus improving the intestinal absorption of amino acid in animals. Condensed tannins in several plants used as animal food such as *Lotus corniculatus* and *Hedysarum coronarium* have been shown to be beneficial for ruminants and have contributed to the production of more milk, increased growth of wool, ovulation rate, and lambing percentage while lessening the risk of bloating and also decreasing the burden of parasites. This is likely to be associated with increased absorption of essential amino acids from the small intestine by condensed tannins. Owing to their astringent nature, tannins are known to be constituents of several drugs. They are used in the management and cure of hemorrhoids, diarrhoa, dysentery, and leucorrhoea and as a helpful pharmaceutical for throat infections (190). Flavonoid medications have been widely used to ameliorate circulatory disorders involving capillary dysfunction. They are also known to be efficient in averting and mitigating capillary fragility and permeability (191). Legumes contain primary and secondary metabolites and other phytochemicals such as nutraceuticals, pharmaceuticals, pesticides, and industrial products (192). The use of natural products, particularly plants, for the treatment of a variety of disorders is as old as mankind (193). Numerous researchers documented that antimicrobial activity is associated with plant secondary metabolites such as saponins, tannins, alkaloids, flavonoids, quinines, and phenolic compounds (194–196). Alkaloids have been used for skin infection treatments (196, 197).

## 6. Conclusion and future directions

ANFs are widely found in daily foods and have been laced with tremendous health benefits. Vast literature supports the negative impacts of ANFs on human health and little emphasis is placed on the beneficial effects of ANFs. Growing evidence as discussed in this review article clearly indicates that regulated consumption of ANFs may help to overcome a wide range of human diseases and are beneficial for regulating several physiological and metabolic processes. While many of the potentially harmful compounds from plants have been isolated and explored, less significance has been given to the positive impact when compared to their antinutritive or interfering effects. As plant breeders and nutritionists are finding ways to minimize the presence of these antinutrients in plant-based foods, endeavors should be aimed at maximizing the salutary and therapeutic worth of these entities. Chemically synthesized drugs are costly, and micro-organisms are developing resistance to them in the form of drug resistance. In this regard, it is worth exploring natural plant-based compounds to tackle the issue of drug resistance encountered in the management and treatment of various diseases. We believe that the current account on the positive benefits of ANFs will provide a strong platform for promoting future investigation and exploration of ANFs to devise proper strategies to incorporate them and use them as therapeutic tools for human aliments.

## Author contributions

RS, IBN, OMB, SA, AT, and RAM: conceptualization. RS, IBN, OMB, SA, AT, and RAM: writing—original draft preparation. OMB, SA, AT, and RAM: review and editing. All authors have read and agreed to the published version of the manuscript.

## Conflict of interest

The authors declare that the research was conducted in the absence of any commercial or financial relationships that could be construed as a potential conflict of interest.

## Publisher’s note

All claims expressed in this article are solely those of the authors and do not necessarily represent those of their affiliated organizations, or those of the publisher, the editors and the reviewers. Any product that may be evaluated in this article, or claim that may be made by its manufacturer, is not guaranteed or endorsed by the publisher.
